# Endogenous GLP-1 levels play an important role in determining the efficacy of DPP-IV Inhibitors in both prediabetes and type 2 diabetes

**DOI:** 10.3389/fendo.2022.1012412

**Published:** 2022-10-04

**Authors:** Shiau Chin Chong, Norlela Sukor, Sarah Anne Robert, Kim Fong Ng, Nor Azmi Kamaruddin

**Affiliations:** ^1^ Department of Medicine, Universiti Kebangsaan Malaysia Medical Centre (UKMMC), Kuala Lumpur, Malaysia; ^2^ Department of Pharmacy, Universiti Kebangsaan Malaysia Medical Centre (UKMMC), Kuala Lumpur, Malaysia; ^3^ Department of Cardiology, Hospital Sultanah Aminah Johor Bahru, Johor, Malaysia

**Keywords:** dipeptidyl peptidase-IV inhibitor, efficacy, glucagon-like peptide-1, linagliptin, prediabetes, type 2 diabetes

## Abstract

**Background:**

In contrast to Western population, glucagon-like peptide-1 (GLP-1) levels are preserved in some East Asian population with type 2 diabetes (T2D), explaining why dipeptidyl peptidase-IV (DPP-IV) inhibitors are more effective in East Asians. We assessed whether differences in endogenous GLP-1 levels resulted in different treatment responses to DPP-IV inhibitors in prediabetes and T2D.

**Methods:**

A prospective 12-week study using linagliptin 5mg once daily in 50 subjects (28 prediabetes and 22 T2D) who were stratified into high versus low fasting GLP-1 groups. A 75-g oral glucose tolerance test (OGTT) was performed at week 0 and 12. Primary outcomes were changes in HbA1c, fasting and post-OGTT glucose after 12 weeks. Secondary outcomes included changes in insulin resistance and beta cell function indices.

**Results:**

There was a greater HbA1c reduction in subjects with high GLP-1 compared to low GLP-1 levels in both the prediabetes and T2D populations [least-squares mean (LS-mean) change of -0.33% vs. -0.11% and -1.48% vs. -0.90% respectively)]. Linagliptin significantly reduced glucose excursion by 18% in high GLP-1 compared with 8% in low GLP-1 prediabetes groups. The reduction in glucose excursion was greater in high GLP-1 compared to low GLP-1 T2D by 30% and 21% respectively. There were significant LS-mean between-group differences in fasting glucose (-0.95 mmol/L), 2-hour glucose post-OGTT (-2.4 mmol/L) in the high GLP-1 T2D group. Improvement in insulin resistance indices were seen in the high GLP-1 T2D group while high GLP-1 prediabetes group demonstrated improvement in beta cell function indices. No incidence of hypoglycemia was reported.

**Conclusions:**

Linagliptin resulted in a greater HbA1c reduction in the high GLP-1 prediabetes and T2D compared to low GLP-1 groups. Endogenous GLP-1 level play an important role in determining the efficacy of DPP-IV inhibitors irrespective of the abnormal glucose tolerance states.

## 1 Introduction

Glucagon-like peptide-1 (GLP-1) is an incretin hormone which plays an important role in the physiological regulation of glucose homeostasis. GLP-1 is rapidly inactivated by the enzyme dipeptidyl peptidase-IV (DPPIV). To overcome the very short half-life of GLP-1, DPP-IV inhibitors have been developed. The enhancement of active endogenous GLP-1 leads to increased insulin but reduced glucagon secretion, in addition to delaying gastric emptying and supressing appetite (1). Linagliptin is an oral, once-daily selective DPP-IV inhibitor. Treatment with linagliptin monotherapy or in combination with other oral glucose lowering drugs (OGLDs) produces clinically significant improvement in glycaemic control among type 2 diabetes (T2D) patients (2, 3). In addition, linagliptin has been shown to be effective in reducing glucose excursion in prediabetes (4, 5).

Pathophysiology of T2D is characterized by predominant insulin resistance than impaired insulin secretion in Caucasians whereas impaired insulin secretion rather than insulin resistance predominates in Asians (6). Given the growing body of evidence that the pathophysiology of T2D differs by ethnic groups, a number of systematic reviews and meta-analyses have been conducted to examine ethnic differences in the glucose-lowering efficacy of DPP-IV inhibitors (2, 3, 7–10). These analyses included large-scale, multi-regional studies which enrolled patients from either predominantly Asian (11) or Western (12, 13) countries. Kim et al. (7) revealed that DPP-IV inhibitors lowered HbA1c to a greater magnitude in studies with ≥50% Asian participants than in studies with<50% Asian participants. The between-group difference was −0.26% (95% confidence interval [CI] −0.36 to −0.17%, p< 0.001). The overall decrease in fasting plasma glucose (FPG) was significantly larger in the Asian-dominant studies than in non-Asian-dominant studies; the difference between the two groups was −0.45 mmol/L (95% CI −0.79 to −0.10 mmol/L). When a more robust ethnic-specific population cut-off of 70% was used, Gan et al. (9) also found -0.11% (p = 0.0098) greater reduction in HbA1c in the predominantly Asian compared to the predominantly Caucasian populations. Similar findings were found by Park et al. (8) where the HbA1c reduction with DPP-IV inhibitors was more than doubled in Japanese-specific trials compared to non-Japanese trials (weighted mean difference –1.67% versus – 0.66%, p< 0.05). HbA1c reduction at each trough DPP-IV inhibition level was larger in Japanese than non-Japanese patients (10).

It is apparent that the therapeutic effects of DPP-IV inhibitors in Asians are more pronounced compared with Caucasians. Regional differences in environment, pharmacogenetic, lifestyle and dietary differences of the patients might affect their treatment responses. Previous studies have suggested that deficiency in GLP-1 secretion partly contribute to the diminished incretin effect typically observed among Caucasians with T2D (14). However, recent studies conducted in East Asia revealed that GLP-1 levels are not reduced in T2D patients (15). Hence, question has been raised whether difference in GLP-1 levels between Asian and Caucasian populations has the potential to explain the difference in responses to DPP-IV inhibitors. Although extensive research has been carried out to identify clinical characteristics of patients that predict the therapeutic efficacy of DPP-IV inhibitor (16–18), very little attention has been paid to the role of basal (fasting) endogenous GLP-1 level in mediating the therapeutic effect of DPP-IV inhibitor. In fact, previous research have examined the mechanism of action of sitagliptin (19) and vildagliptin (20) underlying the improved glucose control. However, individual DPP-IV inhibitor differs in its pharmacokinetic or pharmacodynamic properties. Therefore, this study aimed to examine whether differences in basal endogenous GLP-1 levels in prediabetes and T2D patients affected their responses to linagliptin treatment. The primary outcome in this study were changes from baseline in HbA1c, FPG and glucose responses following an oral glucose tolerance test (OGTT) after 12 weeks of linagliptin treatment. The secondary outcomes included changes from baseline in markers of insulin resistance, insulin sensitivity and beta cell function during an OGTT.

## 2 Materials and methods

### 2.1 Study participants

This was a prospective study conducted over a 12-week period. Patients who attended their routine health examination were invited for OGTT screening. Prediabetes and T2D were diagnosed according to the American Diabetes Association Guidelines (21). Prediabetes was defined as having impaired fasting glucose and/or impaired glucose tolerance. We also recruited subjects diagnosed with T2D from the specialized diabetes clinics. Eligible subjects included 18 to 75 years of age, HbA1c ≥6.5% and ≤10%, T2D patients for at least 3 months on stable doses of either metformin, sulphonylurea, alpha-glucosidase inhibitor or combination therapy. Subjects were excluded if they had anemia (hemoglobin<10 g/dL), renal dysfunction (serum creatinine >130 μmol/L), elevated liver enzymes (twice the upper limit of normal values), chronic lung disease; or if they had myocardial infarction, stroke or transient ischemic attack within 6 months; or those who were pregnant or breastfeeding; or if they had received DPP-IV inhibitor, GLP-1 analogue or insulin treatment within the prior 3 months.

The study was approved by the local ethics committees and conformed to the declaration of Helsinki. Written informed consent was obtained from all subjects.

### 2.2 Methods

#### 2.2.1 Study procedure

After an overnight fast of 10 hours at weeks 0 and 12, fasting blood samples were collected for baseline hematological and biochemical assessments. Anthropometric measurements and vital signs were obtained. For subjects with T2D, the last 2 doses of OGLDs were withheld prior to the study. Each subject then ingested a 75-g glucose within 5 minutes. Venous blood samples were drawn at times 0 (before initiation of OGTT), 30, 60, 90, 120 minutes from the indwelling catheter for measurement of glucose and insulin. All prediabetes and T2D subjects received linagliptin 5mg once daily for a duration of 12 weeks. During the treatment period, T2D subjects were allowed to continue their existing, stable background regimen of OGLDs. All subjects were required to attend study visit at week 6 during which compliance was assessed by pill counting. Subjects whose compliance< 80% were discontinued from the study. At week 12, the study medication was taken 30 min before the repeated OGTT. Fasting plasma samples for total GLP-1 determination were collected at week 0. HbA1c was measured at week 0 and 12. All adverse events related to the study medication were monitored throughout the study and documented.

#### 2.2.2 Laboratory analyses

Fasting plasma samples for total GLP-1 measurement were collected in an EDTA tube without any aprotinin, DPP-IV inhibitor or anticoagulant. The tubes were kept on ice until centrifugation. After centrifugation at 3000 g for 15 min at 4 ^0^C, the samples were stored in aliquots at -80^0^C until further analysis. The total GLP-1 (7-36 and 9-36) were determined by ELISA (EMD Millipore, Billerica, MA, USA). HbA1c was measured using an automatic HbA1c analyzer (D10, Bio-Rad, California, USA). Plasma glucose, total cholesterol, HDL cholesterol and triglyceride levels were analyzed by a Roche Cobas 8000 modular analyzer (Basel, Switzerland). LDL cholesterol was calculated using Friedewald’s formula (22). The serum insulin was measured by an electrochemiluminescence immunoassay (Cobas E411, Roche, Switzerland).

#### 2.2.3 Definitions

To examine the effect of fasting total GLP-1 level on the response to linagliptin during an OGTT, prediabetes and T2D subjects were divided into high GLP-1 and low GLP-1 groups based on the median of fasting GLP-1 levels. Individuals above the median GLP-1 level were categorised as high GLP-1 group whereas those below the median level as low GLP-1 group.

The area under the curve (AUC) for glucose and insulin were estimated according to the trapezoidal rule for 0- to 120-minute during the OGTT. Glucose excursion was defined as the percent change in glucose concentration (AUC_Glucose_) from baseline to week-12 (23).

Indices of insulin resistance at fasting state:

Homeostasis model assessment of insulin resistance (HOMA-IR):


$${\text{HOMA-IR=(fasting insulin (μUml}}^{-1})\times \text{fasting glucose}\;\text{(mmol}\;{\text{l}}^{-1})/22.5)$$

(24)

Triglyceride-glucose (TyG) index:


$$\text{TyG=(ln[fasting}\;\text{glucose}\;{\text{(mgdL}}^{-1})\;\times \;\text{triglyceride}\;{\text{(mgdL}}^{-1})/\;2]$$

(25)

Homeostasis resistance of insulin sensitivity (HOMA-IS) was used to evaluate insulin sensitivity at fasting state:m


HOMA-IS = (1/HOMA - IR)

(26)

Homeostasis model assessment of beta cell function (HOMA-β) was used to assess insulin secretory capacity of pancreatic beta cell at fasting state:


$$\text{HOMA-β=(20}\;\times \;\text{fasting}\;\text{insulin}\;{\text{(mUL}}^{-1}\text{))/(fasting}\;\text{glucose}\;\text{(mmol}\;{\text{l}}^{-1})\;-\;3.5)$$

(27)

Insulinogenic index was used as a measurement of early phase insulin response to OGTT:


$$\text{Insulinogenic index =}\;{\text{(Δinsulin}}_{0-30}{\text{/Δglucose}}_{0-30})$$

(28)

Oral disposition index was used to provide an estimate of beta cell function relative to the prevailing insulin resistance level during the OGTT:


$$\text{Oral}\;\text{disposition}\;\text{index}\;\text{(Δ}\;{\text{insulin}}_{0-30}{\text{/Δglucose}}_{0-30}\text{)/fasting}\;\text{insulin)}$$

(29)

### 2.3 Statistical analyses

Based on sample size calculation, a minimum of 32 subjects were needed for this study, taking into account 10% drop out rate, standard deviation of 1.59%, with 90% power to detect the difference of at least 2.1% in glucose level at 120 min during an OGTT between high GLP-1 and low GLP-1 groups (two-sided test, α = 0.05).

Data were analysed using SPSS for windows version 23 (Chicago, IL, USA). All continuous variables were expressed as the mean ± SD or as median (25^th^ percentile, 75^th^ percentile) if the data were not normally distributed. Differences between the baseline and after 12-week of treatment were analysed using paired t-test or Wilcoxon signed-rank test. Between-group comparisons were made by independent Student’s t-test or Mann-Whitney test. The chi-square test was used for categorical variables. The ANCOVA model was used to compare least-squares mean (LS-mean) change from baseline value at week 12 with covariates included baseline value, ethnicity and concomitant OGLDs.

## 3 Results

### 3.1 Patient demography and clinical characteristics

**Figure 1:**
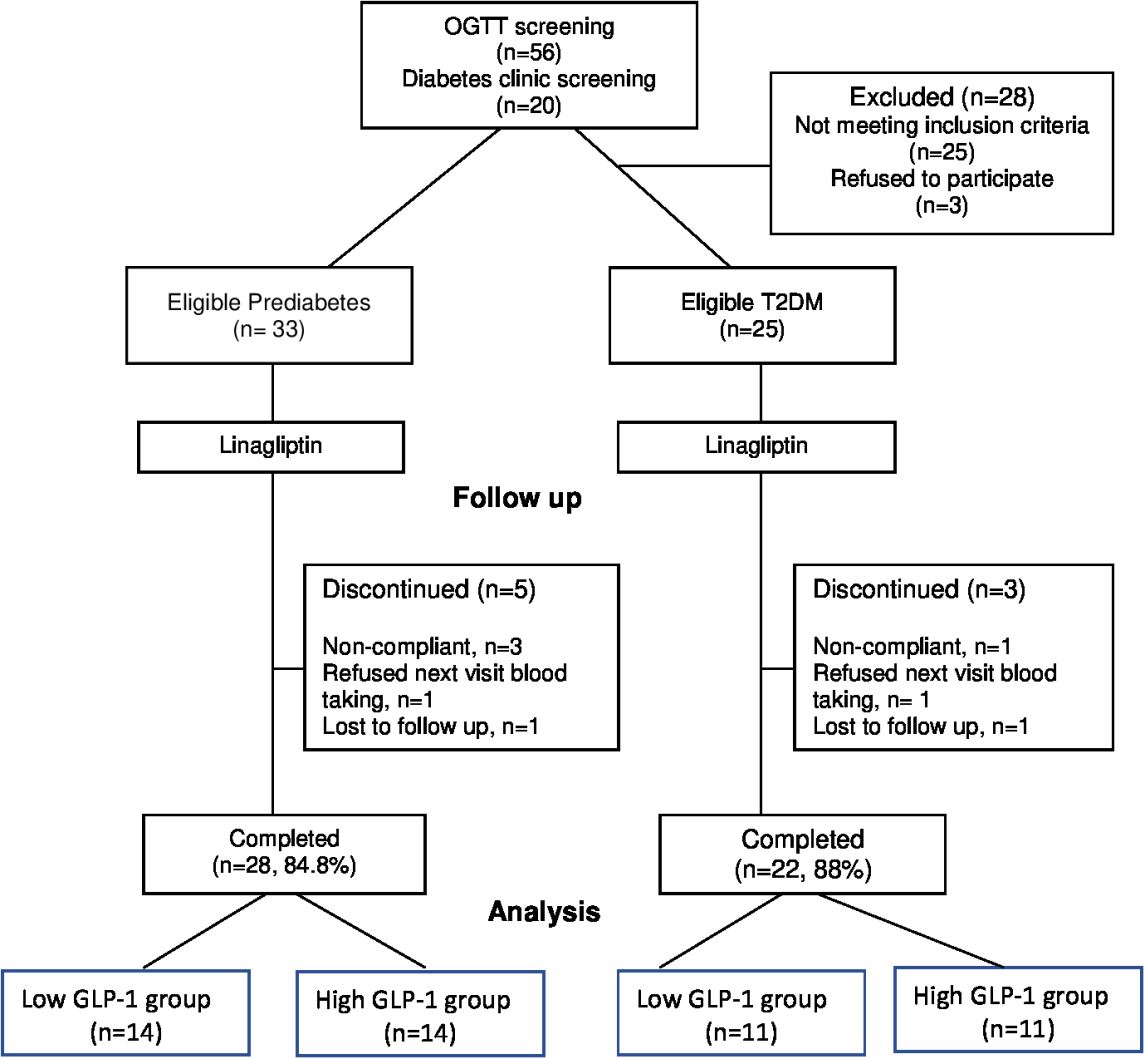
Patient disposition.

A total of 76 subjects were screened but only 58 subjects (33 prediabetes and 25 T2D) subjects met the study criteria and consented to be enrolled in the study (**Figure 1**). All subjects were given linagliptin 5 mg daily in the morning for 12 weeks. At the end of the 12-week, 50 subjects (28 prediabetes and 22 T2D) completed the study. Among the T2D subjects, 8 were newly diagnosed and 14 subjects were known diabetes with a mean duration of 3.2 ± 2.8 years (mean ± SD). They were treated with a minimum 3-month stable doses of OGLDs (metformin [n = 3], sulphonylurea [n = 1], metformin plus sulphonylurea [n = 7] as well as metformin plus sulphonylurea plus acarbose [n = 3]). Prediabetes subjects were divided into low GLP-1 group (<21.11 pmol/L) and high GLP-1 group (>21.11 pmol/L) based on the median of fasting total GLP-1 levels measured before the OGTT at week 0. Similarly, T2D subjects were categorized into two groups by the median (26.07 pmol/L) of their fasting total GLP-1 levels.

The baseline demography, anthropometry, clinical and biochemical parameters were similarly distributed between the prediabetes and T2D subjects, with the exception of T2D subjects who had significantly higher FPG and HbA1c than prediabetes as expected (**Table 1**). In general, there were no significant differences in the baseline characteristics between low versus high GLP-1 groups in both prediabetes and T2D populations (**Table 2**). The median fasting GLP-1 in the low versus high GLP-1 groups in the prediabetes and T2D subjects were 16.06 pmol/L versus 27.28 pmol/L and 21.37 pmol/L versus 30.39 pmol/L respectively. This reflected different cut-off values of fasting GLP-1 levels employed in defining low and high GLP-1 groups.

**Table 1:** Baseline demography and clinical characteristics of prediabetes versus type 2 diabetes.

	Prediabetes (n=28)	Type 2 diabetes (n=22)	p-value
Age (years)	50 ± 10	52 ± 12	0.484
Male/Female, %	50/50	72.7/27.3	0.09
Ethnicity, n (%) Malays Chinese Indians	9 (32.1) 11 (39.3) 8 (28.6)	8 (36.4) 8 (36.4) 6 (27.3)	0.951
Body weight (kg)	73.99 ± 13.26	75.09 ± 15.26	0.787
Height (cm)	161.30 ± 8.23	163.64 ± 9.27	0.352
BMI (kg/m^2^)	28.42 ± 4.65	28.16 ± 6.10	0.868
Waist-to-hip ratio	0.90 ± 0.09	0.92 ± 0.07	0.469
Systolic blood pressure (mmHg)	135 ± 18	134 ± 18	0.849
Diastolic blood pressure (mmHg)	82 ± 11	80 ± 10	0.712
HbA1c (%)	5.95 ± 0.45	8.32 ± 0.84	<0.001
Fasting glucose (mmol/L)	5.46 ± 0.77	9.12 ± 2.08	<0.001
Fasting insulin (µU/ml)	18.15 ± 7.94	18.82 ± 13.89	0.839
Fasting GLP-1 (pmol/L)	21.11 (15.77, 27.53)	26.09 (21.27, 31.48)	0.044
Triglyceride (mmol/L)	1.60 (1, 2.03)	1.65 (1.3, 2.23)	0.252
Total cholesterol (mmol/L)	4.83 ± 0.98	5.15 ± 1.42	0.344
LDL-C (mmol/L)	2.74 ± 0.69	3.12 ± 1.26	0.209
HDL-C (mmol/L)	1.18 ± 0.35	1.09 ± 0.18	0.249

Data are expressed as mean ± standard deviation or median (25^th^ percentile, 75^th^ percentile).

**Table 2:** Baseline demography and clinical characteristics of low versus high GLP-1 levels in prediabetes and type 2 diabetes subjects.

	Prediabetes (n=28)			Type 2 diabetes (n=22)		
	Low GLP-1 (n=14)	High GLP-1 (n=14)	p-value	Low GLP-1 (n=11)	High GLP-1 (n=11)	p-value
Age (years)	52 ± 10	49 ± 10	0.344	56 ± 13	49 ± 10	0.127
Male/Female, %	35.7/64.3	64.3/35.7	0.111	72.7/27.3	72.7/27.3	1.000
Ethnicity, n (%)						
**Malays**	4 (28.6)	5 (35.7)	1.000	3 (27.3)	5 (45.5)	0.861
**Chinese**	6 (42.9)	5 (35.7)		5 (45.5)	3 (27.3)	
**Indians**	4 (28.6)	4 (28.6)		3 (27.3)	3 (27.3)	
Body weight (kg)	71.69 ± 15.33	76.29 ± 10.88	0.368	66.3 (61.4,91.3)	72.3 (65.5,85.8)	0.341
Height (cm)	159.27 ± 9.77	163.34 ± 6.02	0.199	165.8 ± 9.57	161.47 ± 8.87	0.284
BMI (kg/m^2^)	28.27 ± 5.62	28.56 ± 3.64	0.875	26.48 ± 4.59	25.05 ± 7.13	0.204
Waist-to-hip ratio	0.90 (0.78,0.91)	0.92 (0.88,0.97)	0.241	0.9 ± 0.07	0.93 ± 0.08	0.434
Systolic blood pressure (mmHg)	137 ± 19	132 ± 18	0.452	130 ± 22	136 ± 13	0.475
Diastolic blood pressure (mmHg)	80 ± 10	83 ± 13	0.479	77 ± 11	83 ± 8	0.151
HbA1c (%)	5.94 ± 0.20^c^	5.95 ± 0.62^d^	0.968	8.13 ± 0.96^c^	8.52 ± 0.7^d^	0.288
Fasting glucose (mmol/L)	5.35 ± 0.88^c^	5.57 ± 0.66^d^	0.457	8.85 ± 2.08^c^	9.39 ± 2.14^d^	0.558
Fasting insulin (µU/ml)	17.09 ± 9.41	19.2 ± 6.31	0.493	13.96 (10.88,26.22)	17.71 (10.58,32.62)	0.922
Fasting GLP-1 **(pmol/L)**	16.06^a,c^ (13.7,19.4)	27.28^a^ (24.39,35.85)	<0.001	21.37^b,c^ (19.44,23.67)	30.39^b^ (28,37.32)	<0.001
Triglyceride (mmol/L)	1.3 (0.98,1.7)	1.6 (1.23,2.53)	0.230	1.5 (1.3,1.9)	1.9 (1.2,2.6)	0.921
Total cholesterol **(mmol/L)**	4.7 (4.05,5.3)	4.65 (4.18,5.28)	0.945	4.87 ± 1.4	5.43 ± 1.46	0.373
LDL-C (mmol/L)	2.71 ± 0.7	2.76 ± 0.69	0.851	2.81 ± 1.19	3.44 ± 1.31	0.250
HDL-C (mmol/L)	1.24 ± 0.44	1.13 ± 0.23	0.425	1.07 ± 0.17	1.1 ± 0.19	0.724
Glucose tolerance						
Category, n (%)				3 (21.4)	1 (7.1)	0.276
**IFG**				10 (52.6)	9 (64.3)	
**IGT**				1 (7.1)	4 (28.6)	
**IFG+IGT**						
Duration of diabetes, n (%)						
≤1 year				3 (27.3)	5 (45.5)	0.550
**>1 to<5 years**				2 (18.2)	3 (27.3)	
≥5 years				6 (54.5)	3 (27.3)	
**Oral glucose lowering drugs, n (%)**						
**No medication**				4 (36.4)	3 (27.3)	
**Metformin**				2 (18.2)	2 (18.2)	
**Sulphonylurea**				1 (9.1)	0 ()	
**Metformin+**						1.000
**Sulphonylurea**				3 (37.3)	4 (36.4)	
**Metformin+**						
**Sulphonylurea+**				1 (9.1)	2 (18.2)	
**Acarbose**						

Data are expressed as mean ± standard deviation or median (25^th^ percentile, 75^th^ percentile). IFG, impaired fasting glucose; IGT, impaired glucose tolerance.

a/b/c/d, for each variable, there was significant difference between the groups marked with the same superscript letter.

T2D subjects in the low GLP-1 group had significantly higher fasting GLP-1 level compared to prediabetes with low GLP-1 group. Other metabolic parameters such as total cholesterol, triglyceride, LDL-cholesterol and HDL-cholesterol were not statistically different between the low GLP-1 and high GLP-1 groups.

### 3.2 Glycaemic response

**Figure 2:**
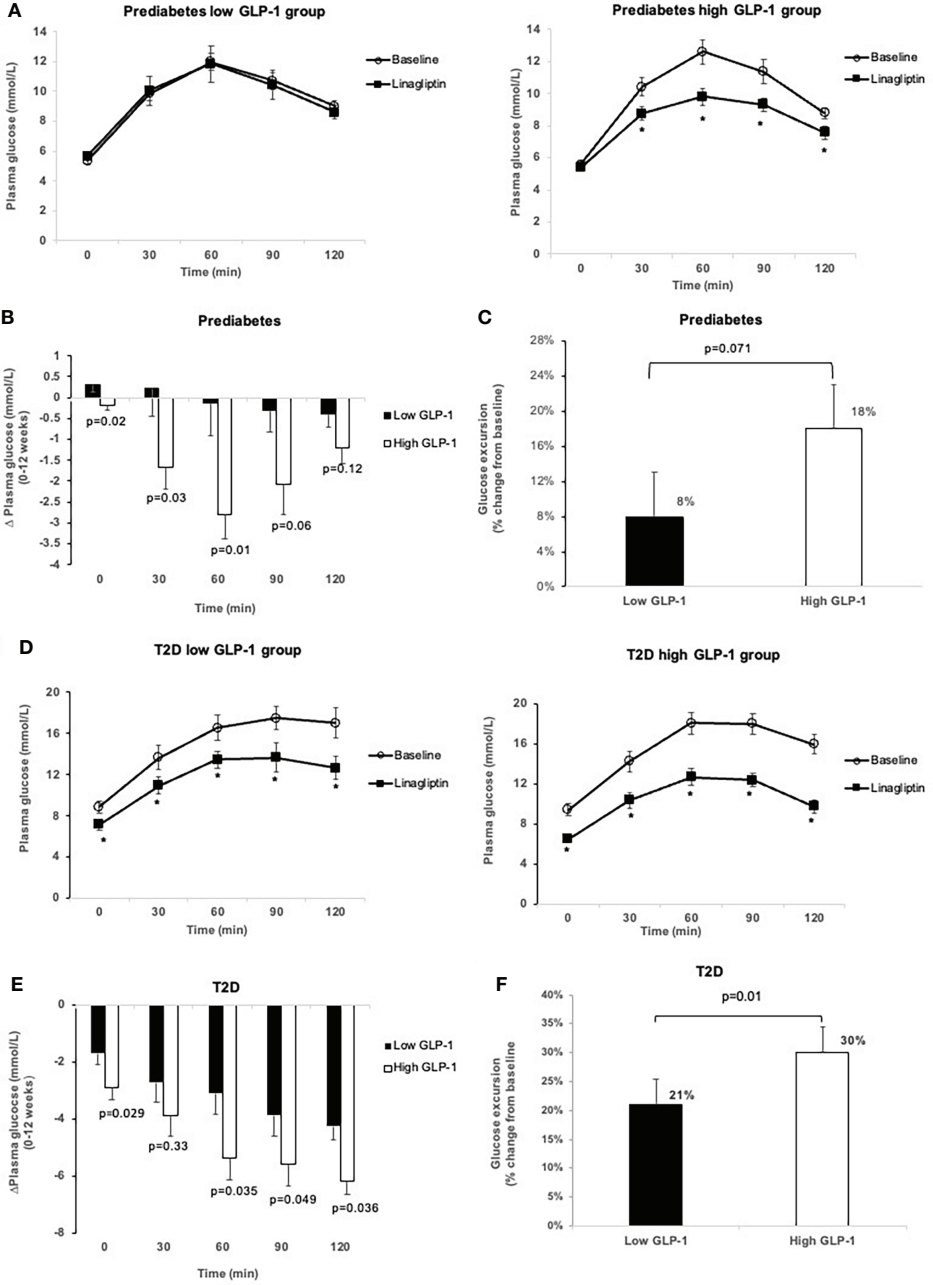
Comparison of glucose levels during OGTT at baseline and after 12-week treatment with linagliptin in low GLP-1 and high GLP-1 groups in prediabetes and T2D: glucose levels in prediabetes **(A)**; change in glucose levels from 0 to 12 weeks in prediabetes **(B)**; glucose excursion in prediabetes **(C)**; glucose levels in T2D **(D)**; change in glucose levels from 0-12 weeks in T2D **(E)**; glucose excursion in T2D **(F)**. White circles: baseline; black squares: after linagliptin treatment; black bars: low GLP-1 group; white bars: high GLP-1 groups. T2D, type 2 diabetes. *p<0.05. Data shown are mean ± standard error.

In the prediabetes group, treatment with linagliptin 5 mg once-daily for 12 weeks significantly reduced HbA1c from baseline in the high GLP-1 group [LS-mean change from baseline -0.33% (95% CI -0.48, -0.18). This resulted in a significant greater reduction in HbA1c at week 12 in the high GLP-1 group compared to the low GLP-1 group [between-group difference -0.22% (95% CI -0.43, -0.02)] (**Table 3**). Treatment with linagliptin did not change the FPG in the high GLP-1 group. However, the LS-mean change in FPG from baseline between the low and high GLP-1 groups was significantly differed by -0.45 mmol/L (95% CI -0.78, -0.12). Linagliptin lowered plasma glucose levels significantly at 30, 60, 90 and 120-minute during OGTT in high GLP-1 group (**Figures 2A, B** **)**. Moreover, there was a significant reduction in LS-mean change in AUC_Glucose0-2h_ from baseline across the high GLP-1 group [LS-mean change from baseline -263.89 mmol/L (95% CI -384.74, -143.04)]. There was also a significant reduction in AUC_Glucose0-2h_ in the high GLP-1 compared to the low GLP-1 group at 12 weeks [between group difference -172.24 mmol/L.min (95% CI -343.53,-0.95)] (**Table 3**).

**Table 3:** Changes in HbA1c, plasma glucose and serum insulin in prediabetes and type 2 diabetes.

Prediabetes	n	Baseline (mean ± SD)	Week 12 (mean ± SD)	LS-mean change from baseline (95% CI)	LS-mean between- group difference (95% CI)
**HbA1c (%)**					
**Low GLP-1**	14	5.94 ± 0.20	5.84 ± 0.31	-0.11 (-0.26,0.04)	-0.22 (-0.43,-0.02)*
**High GLP-1**	14	5.95 ± 0.62	5.62 ± 0.62	-0.33 (-0.48,-0.18)^†^	
**Fasting plasma glucose (mmol/L)**					
**Low GLP-1**	14	5.35 ± 0.88	5.67 ± 0.68	0.29 (0.05,0.52)	-0.45 (-0.78,-0.12)*
**High GLP-1**	14	5.57 ± 0.66	5.37 ± 0.50	-0.16 (-0.39,0.07)	
**2-h plasma glucose (mmol/L)**					
**Low GLP-1**	14	9.01 ± 1.31	8.61 ± 1.78	-0.38 (-1.1,0.37)	-0.85 (-1.92,0.22)
**High GLP-1**	14	8.77 ± 1.18	7.57 ± 1.56	-1.23 (-1.99,-0.48)^†^	
**Glucose AUC_0-2h_ (mmol/L.min)**					
**Low GLP-1**	14	1256.57 ± 232.97	1181.36 ± 359.47	-91.65 (-213.42,30.12)	-172.24* (-343.53, -0.95)
**High GLP-1**	14	1294.39 ± 289.48	1028.25 ± 146.70	-263.89† (-384.74, -143.04)	
**Fasting insulin (µU/ml)**					
**Low GLP-1**	14	17.09 ± 9.41	15.16 ± 8.84	-1.74 (-3.69,0.21)	1.10 (-1.66,3.87)
**High GLP-1**	14	19.20 ± 6.31	18.23 ± 5.67	-0.63 (-2.58,1.31)	
**30- min insulin (µU/ml)**					
**Low GLP-1**	14	61.32 ± 38.09	66.81 ± 35.96	-2.57 (-16.31,11.18)	31.13 (9.94,52.33)*
**High GLP-1**	14	117.74 ± 44.40	136.53 ± 35.45	28.57^†^ (15.21,41.92)	
**Insulin AUC_0-2h_ (µU/ml.min)**					
**Low GLP-1**	14	10011.01±5495.12	10350.56±4481.95	517.45 (-2854.14,3889.05)	2489.1 (-2741.39,7719.59)
**High GLP-1**	14	18539.53±6493.01	20973.60±9348.39	3006.55 (-253.42,6266.53)	
**Type 2 diabetes**	**n**	**Baseline** **mean ± SD**	**Week 12** **mean ± SD**	**LS-mean change from baseline** **(95% CI)**	**LS-mean between- group difference** **(95% CI)**
**HbA1c (%)**					
**Low GLP-1**	11	8.13 ± 0.96	7.35 ± 0.75	-0.9 (-1.22,-0.58)^†^	-0.58 (-1.04,-0.12)*
**High GLP-1**	11	8.52 ± 0.70	6.93 ± 0.39	-1.48 (-1.8,-1.16)^†^	
**Fasting plasma glucose (mmol/L)**					
**Low GLP-1**	11	8.85 ± 2.08	7.16 ± 1.86	-1.85 (-2.46,-1.25)^†^	-0.95 (-1.82,-0.08)*
**High GLP-1**	11	9.39 ± 2.14	6.48 ± 1.29	-2.81 (-3.42,-2.19)^†^	
**2-h plasma glucose (mmol/L)**					
**Low GLP-1**	11	17.03 ± 4.79	12.66 ± 3.81	-4.02 (-5.08,-2.95)^†^	-2.40 (-3.94,-0.87)*
**High GLP-1**	11	15.93 ± 3.11	9.74 ± 2.18	-6.42 (-7.48,-5.35)^†^	
**Glucose AUC_0-2h_ (mmol/L.min)**					
**Low GLP-1**	11	1826.59 ± 460.36	1427.73 ± 297.86	-410.3† (-492.09,-328.51)	-156.26* (-272.81,-39.72)
**High GLP-1**	11	1886.59 ± 369.96	1303.91 ± 241.94	-566.56^†^ (-648.39,-484.74)	
**Fasting insulin (µU/ml)**					
**Low GLP-1**	11	18.79 ± 11.70	18.70 ± 15.48	-0.12 (-8.40,8.16)	0.92 (-10.88,12.72)
**High GLP-1**	11	18.85 ± 16.38	19.58 ± 13.11	0.80 (-7.49,9.09)	
**30- min insulin (µU/ml)**					
**Low GLP-1**	11	48.08 ± 22.29	49.13 ± 26.17	0.17 (-10.95,11.29)	8.72 (-7.18,24.62)
**High GLP-1**	11	60.37 ± 43.87	69.85 ± 41.23	8.89 (-2.09,19.87)	
**Insulin AUC_0-2h_ (µU/ml.min)**					
**Low GLP-1**	11	7479.22±3823.06	8474.23±4084.95	2045.02^†^ (62.26,4027.77)	3064.55* (160.28,5968.81)
**High GLP-1**	11	13555.91±9709.1	19708.59±13918.79	5109.56^†^ (3148.9,7070.23)	

AUC, area under the curve; GLP-1, glucagon-like peptide-1; LS, least-squares.

*p<0.05; ^†^p<0.05 compared to baseline.

Treatment with linagliptin did not change the FPG and 30-min plasma glucose in the low GLP-1 group with prediabetes (**Figures 2A, B** **)**. The low GLP-1 group only had small but no significant decrease in glucose levels at 60, 90 and 120-minute during OGTT. Overall, linagliptin significantly reduced the OGTT-associated glucose excursion by 18% (95% CI -26.55, -10.34) in the high GLP-1 group compared with 8% (95% CI -16.09 to 0.24) in the low GLP-1 group (**Figure 2C**). The between-group difference approached statistical significance (p = 0.071).

In T2D group, linagliptin significantly reduced HbA1c from baseline in both the low GLP-1 and high GLP-1 groups. The LS-mean reduction in HbA1c in the high GLP-1 was significantly greater than in the low GLP-1 groups by -0.58% (95% CI -1.04, -0.12%). Linagliptin significantly decreased fasting and postload plasma glucose levels in both the low and high GLP-1groups (**Table 3** **;** **Figures 2D, E** **)**. The LS-mean change in FPG from baseline between the low and high GLP-1 groups was significantly differed by -0.95 mmol/L (95% CI -1.82, -0.08). For the LS-mean change in 2-h OGTT plasma glucose from baseline, the between-group difference was -2.4 mmol/L (95% CI -3.94, -0.87) (**Table 3**). There was a significant reduction in LS-mean change from baseline in AUC_Glucose0-2h_ at 12 weeks both in the high GLP-1 and low GLP-1 groups with a greater degree of reduction seen in the high GLP-1 group compared to the low GLP-1 group by -156.26 mmol/L.min (95% CI -272.81, -39.72 mmol/L.min). This was associated with a significant reduction in the glucose excursion by 30% (95% CI -34.53, -25.45) in the high GLP-1 and 21% (95% CI -25.62, -16.55) in the low GLP-1 group, giving rise to a significant between-group difference (p = 0.01) (**Figure 2F**).

### 3.3 Insulin response

**Figure 3:**
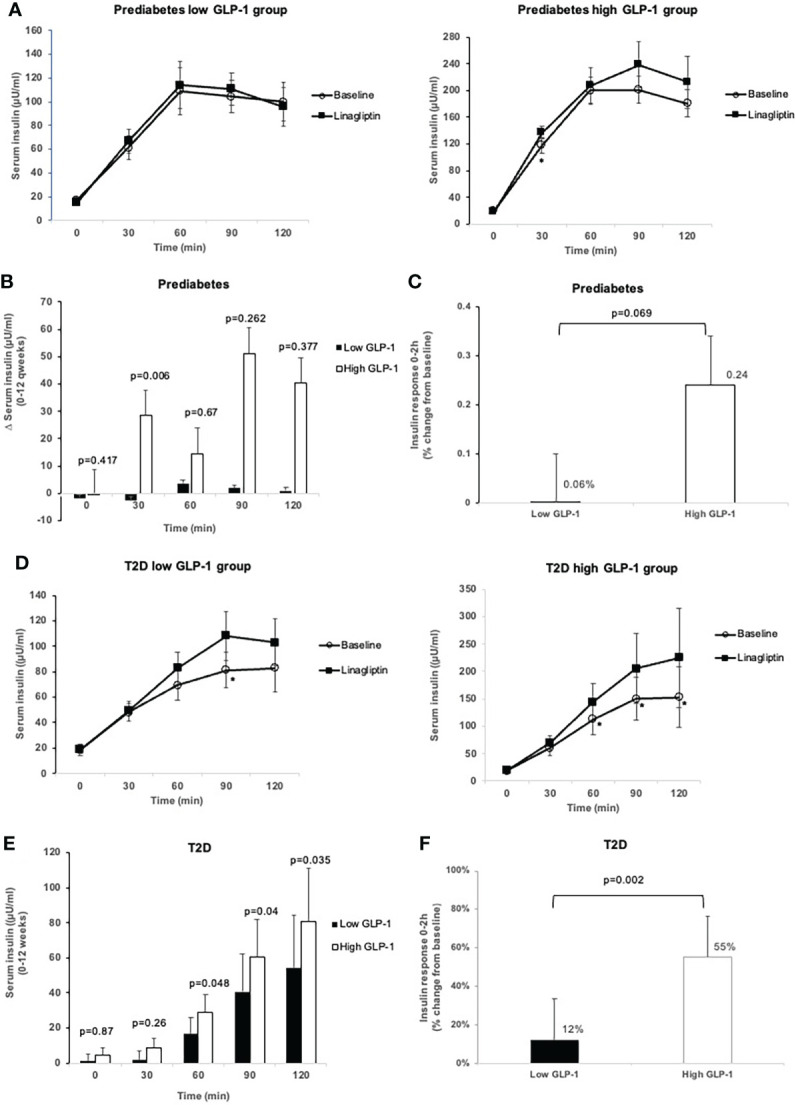
Comparison of insulin levels during OGTT at baseline and after 12-week treatment with linagliptin in low GLP-1 and high GLP-1 groups in prediabetes and T2D: Insulin level at each time point in prediabetes **(A)**; change in insulin levels from 0 to 12 weeks in prediabetes **(B)**; insulin response 0-2h (percent change from baseline) in prediabetes **(C)**; insulin level at each time point in T2D **(D)**, change in insulin levels from 0-12 weeks in T2D **(E)**; insulin response 0-2h (percent change from baseline) in T2D **(F)**. White circles: baseline; black squares: after linagliptin treatment; black bars: low GLP-1 group; white bars: high GLP-1 groups. T2D, type 2 diabetes. *p<0.05. Data shown are mean ± standard error.

In the prediabetes group, treatment with linagliptin did not alter fasting and postload serum insulin levels in prediabetes in both the low and high GLP-1 groups (**Table 3**, **Figures 3A–C**). However, in the high GLP-1 group, the change from baseline in the early (30-minute) LS-mean insulin response to OGTT was increased significantly at 12 weeks of treatment compared to the low GLP-1 groups [between group difference 31.13 µU/ml (95% CI 9.94, 52.33 µU/ml).

In T2D patients with low GLP-1, linagliptin significantly increased serum insulin from baseline at 90-minute during the OGTT (**Figure 3D**). There was a significant increase from baseline in insulin levels at 60, 90 and 120 minutes in high GLP-1 T2D group (**Figure 3D**), resulting in significantly greater LS-mean increment from baseline in these insulin levels in high GLP-1 compared with low GLP-1 groups (**Figure 3E**). Linagliptin significantly increased LS-mean insulin response to OGTT by 55% (95% CI 39.05, 71.74%) in the high GLP-1 compared to 12% (95% CI -4.05, 29.01%) in low GLP-1 group, resulted in a significant between-group difference (p = 0.002) (**Figure 3F**).

### 3.3 Insulin resistance (HOMA-IR, TyG index) and beta cell function indices

Linagliptin resulted in a reduction of HOMA-IR in the high GLP-1 prediabetes subjects, albeit not significant. On the other hand, TyG index was significantly improved in the high GLP-1 prediabetes group treated with linagliptin [LS-mean change from baseline -0.195 (95% CI -0.33, -0.06). This resulted in a significantly greater improvement in the TyG index at week 12 in the high GLP-1 group compared to the low GLP-1 group [between-group difference -0.19 (95% CI -0.39, -0.01)] (**Table 4**).

**Table 4:** Change from baseline in indices of insulin resistance and beta cell function in subjects with prediabetes and type 2 diabetes.

Prediabetes	n	Baseline mean ± SD	Week 12 mean ± SD	LS-mean change from baseline (95% CI)	LS-mean between- group difference (95% CI)
**HOMA-IR**					
**Low GLP-1**	14	4.17 ± 2.39	3.99 ± 2.15	-0.15 (-0.69,0.38)	-0.19 (-0.95,0.57)
**High GLP-1**	14	4.80 ± 1.80	4.36 ± 1.54	-0.34 (-0.88,0.19)	
**TyG index**					
**Low GLP-1**	14	8.68 ± 0.56	8.67 ± 0.55	-0.002 (-0.14,0.14)	-0.19 (-0.39,-0.01)*
**High GLP-1**	14	8.95 ± 0.50	8.75 ± 0.58	-0.195 (-0.33,-0.06)^†^	
**HOMA-IS**					
**Low GLP-1**	14	0.36 ± 0.27	0.35 ± 0.24	0.001 (-0.07,0.07)	0.023 (-0.08,0.13)
**High GLP-1**	14	0.24 ± 0.12	0.29 ± 0.11	0.024 (-0.05, 0.10)	
**HOMA-β**					
**Low GLP-1**	14	146.42 ± 169.7	151.7 ± 106.04	3.33 (-40.05,33.39)	24.29 (-27.99,76.57)
**High GLP-1**	14	197.14 ± 72.79	203.24 ± 67.68	20.96 (-15.53,57.45)	
**Insulinogenic index**					
**Low GLP-1**	14	11.18 ± 11.11	16.44 ± 11.57	5.63 (-5.20,16.45)	17.9 (1.76,34.04)*
**High GLP-1**	14	21.26 ± 8.75	43.38 ± 25.34	23.53 (13, 34.06)^†^	
**Oral Disposition index**					
**Low GLP-1**	14	0.74 ± 0.6	1.47 ± 1.43	0.89 (-0.13,1.65) 1.17 (0.43,1.91)^†^	0.28 (-0.82,1.38)
**High GLP-1**	14	1.21 ± 0.63	2.48 ± 1.61	1.17 (0.43,1.91)^†^	
**Type 2 diabetes**	**n**	**Baseline** **mean ± SD**	**Week 12** **mean ± SD**	**LS-mean change from baseline** **(95% CI)**	**LS-mean between- group difference** **(95% CI)**
**HOMA-IR**					
**Low GLP-1**	11	7.37 ± 4.57	6.11 ± 5.23	-1.70 (-3.84,0.44)	-3.14 (-6.19,0.10)*
**High GLP-1**	11	8.70 ± 5.21	3.57 ± 3.56	-4.84 (-6.98,-2.71)^†^	
**TyG index**					
**Low GLP-1**	11	9.46 ± 0.62	9.07 ± 0.63	-0.46 (-0.79, -0.12)^†^	-0.57 (-1.05,-0.09)*
**High GLP-1**	11	9.67 ± 0.58	8.59 ± 0.52	-1.03 (-1.36,-0.69)^†^	
**HOMA-IS**					
**Low GLP-1**	11	0.19 ± 0.10	0.28 ± 0.22	0.01 (-1, 1.03)	1.25 (-0.2, 2.70)
**High GLP-1**	11	0.15 ± 0.08	1.21 ± 2.31	1.26 (0.25,2.27)	
**HOMA-β**					
**Low GLP-1**	11	85.28 ± 76.69	131.90 ± 109.81	49.69 (-35.53, 134.91)	36.32 (-85.43,158.07)
**High GLP-1**	11	71.33 ± 58.29	169.70 ± 173.40	86.01 (0.64,171.37)	
**Insulinogenic index**					
**Low GLP-1**	11	10.01 ± 11.58	11.99 ± 12.83	1.83 (-6.64,10.30)	3.93 (-8.15,16.02)
**High GLP-1**	11	11.93 ± 12.95	18.82 ± 21.07	5.76 (-2.70,14.23)	
**Oral Disposition index**					
**Low GLP-1**	11	0.67 ± 0.78	0.87 ± 1.08	0.53 (-4.19,5.24)	3.65 (-3.08,10.39)
**High GLP-1**	11	0.88 ± 1.44	5.52 ± 9.9	4.18 (-0.53,8.89)	

HOMA-β, homeostasis model assessment of beta cell function; HOMA-IR, homeostasis model assessment of insulin resistance; HOMA-IS, homeostasis model assessment of insulin sensitivity; TyG, triglyceride-glucose. *p<0.05; ^†^p<0.05 compared to baseline.

Similarly, treatment with linagliptin resulted in significant improvement in the insulinogenic index in the high GLP-1 prediabetes [LS-mean change from baseline 23.53 (95% CI 13, 34.06)]. There was also a significant increase in the insulinogenic index in the high GLP-1 compared to the low GLP-1 group at 12 weeks [between group difference 17.9 (95% CI 1.76, 34.04)] (**Table 4**). With regards to the oral disposition index, the high GLP-1 group demonstrated significant increment following 12 weeks of treatment with linagliptin [LS-mean change 1.17 (95% CI 0.43, 1.91)].

Linagliptin-treated prediabetes with low GLP-1 level showed small, non-significant decrease in HOMA-IR and TyG index (**Table 4**). Linagliptin appeared to improve HOMA-IS, HOMA-β, insulinogenic index and oral disposition index in low GLP-1 prediabetes although the LS-mean changes from baseline in these indices did not reach statistical significance.

In contrast to prediabetes subjects, linagliptin resulted in a significant reduction in insulin resistance in the high GLP-1 group with T2D (**Table 4**). LS-mean change from baseline in HOMA-IR and TyG index showed significantly greater degree of reduction in high GLP-1 compared with low GLP-1 groups [between group difference -3.14 (95% CI -6.19, -0.100) and between group difference -0.57 (95% -1.05, -0.09) respectively].

## 4 Discussion

The pharmacological action of DPP-IV inhibitors has been studied previously in prediabetes (4, 5) and T2D populations (11–13). However, very little is known about the effects of endogenous GLP-1 levels in determining the efficacy of DPP-IV inhibition in these individuals. In our previous study (submitted for publication) we have shown that prediabetes and T2D subjects have higher GLP-1 levels compared to normal glucose tolerance (30). Hence there is a need to determine if this higher GLP-1 levels in these prediabetes and T2D subjects translate into a better efficacy with DPP-IV inhibition.

To the best of our knowledge, this is the first study ever conducted to investigate the relationship between endogenous GLP-1 levels and glycaemic control as well as insulin responses to DPP-IV inhibition in the form of linagliptin therapy in both prediabetes and T2D subjects. We divided our subjects into low and high GLP-1 groups based on the median fasting GLP-1 levels as the cut-off point. Since GLP-l levels measurement vary with different assays and detection methods, there have been difficulty in deriving the absolute value to define low and high GLP-1. Hence the GLP-1 cut-off remains arbitrary.

Our results demonstrated that the response to DPP-IV inhibitor in prediabetes and T2D is influenced by the baseline endogenous GLP-1 levels as evidenced by the greater reduction in the HbA1c, FPG, AUC_Glucose0-2h_, as well as the glucose excursion seen in the high GLP-1 compared to the low GLP-1 groups in both the prediabetes and T2D populations. In addition, there was a greater reduction in the 2h plasma glucose level in the high GLP-1 compared to the low GLP-1 groups among T2D subjects.

Twelve weeks of linagliptin therapy resulted in a significant reduction in the post-challenge glucose excursion following OGTT (30-minute) in the high GLP-1 subjects with prediabetes. This was also associated with significant improvement in the insulinogenic index in the high GLP-1 prediabetes group. Treatment with linagliptin also yielded small but statistically significant reduction in HbA1c in the high GLP-1 prediabetes group. This modest reduction in HbA1c is probably explained by the modest degree of hyperglycemia present in the prediabetes subjects whose baseline mean HbA1c was only 5.95%.

Our findings highlighted the fact that endogenous baseline GLP-1 concentration has significant influence on insulin secretory and glucose-lowering responses to a larger extent than previously thought. GLP-1 is degraded extensively by the endothelial DPP-IV and consequently only 10-15% of secreted GLP-1 enters the systemic circulation in the active form (31). In general linagliptin has been demonstrated to enhance the systemic concentrations of active GLP-1 by 2-fold (32). This study showcased the magnitude of the therapeutic effect of linagliptin is dependent on the prevailing endogenous GLP-1 levels. When the fasting GLP-1 concentration is comparatively high to begin with, by preventing the proteolytic degradation of GLP-1 with DPP-IV inhibition, considerable increase in GLP-1 responses during the OGTT ensues leading to significant stimulation of insulin secretion and reduction in plasma glucose.

In contrast to the high GLP-1 group, DPP-IV inhibition did not significantly alter plasma glucose, insulin levels, insulin resistance and beta cell function indices in the low GLP-1 prediabetes group. A possible explanation is that DPP-IV inhibition could not raise the GLP-1 levels sufficiently to therapeutic range when the basal GLP-1 level was too low to begin with (33).

The differences in the magnitude of treatment responses to linagliptin between the high and low GLP-1 levels was more pronounced in T2D than prediabetes. This is probably due to the fact that T2D cohort in our study had significantly higher fasting GLP-1 levels compared to prediabetes. This higher fasting GLP-1 level resulted in the higher insulin level during fasting state. In this respect, we postulated that compensatory L-cell secretion of GLP-1 has occurred in our T2D population who were generally still in the early stages of their diabetes (34).

Previous studies have attempted to investigate the potential mechanisms explaining the differences observed in the efficacy of DPP-IV inhibitors between Asians and non-Asians. Our study adds to the growing body of research on the role of basal GLP-1 level where higher level of GLP-1 at fasting state mediates greater treatment response to DPP-IV inhibitor. Other possible mechanisms include enhanced DPP-IV activity (35), lower baseline BMI (<30 kg/m^2^) (16, 36) and increased fish intake (36, 37) in East Asians which contribute to greater therapeutic efficacy of DPP-IV inhibitors. In addition, since the major pathogenesis of T2D in Asians is beta cell dysfunction, incretin-based therapeutic agents exert greater pharmacological actions by ameliorating primary beta cell dysfunction (36, 37).

Linagliptin has been shown to significantly reduced insulin resistance in T2D patients. Previous studies of linagliptin have demonstrated improvement in HOMA-β, insulinogenic index and oral disposition index (3, 11, 12). In this study, the surrogate measures of insulin resistance showed discordant results in the high GLP-1 prediabetes group. While linagliptin did not appreciably alter the HOMA-IR, there was a significant decrease in the TyG index from baseline. Similarly, the TyG index was also significantly lower in the high GLP-1 group compared to the low GLP-1 group in the prediabetes subjects at 12 weeks. TyG index in several recent studies have been shown to be more sensitive than HOMA-IR in determining the insulin resistance and metabolic syndrome state in several population studies (38, 39).

At the end of the study, both HOMA-IR and TyG index were significantly improved in the high GLP-1 group of the T2D population. Similarly, the HOMA-IR and TyG index were significantly lower in the high GLP-1 compared to the lower GLP-1 groups in the T2D population. The improvement in the TyG index and to a lesser extend the HOMA-IR is to be expected as the FPG levels improved significantly following linagliptin intervention in the high GLP-1 group of both the prediabetes and T2D populations despite the non-significant drop in the fasting insulin levels.

We also observed a significant improvement in oral disposition index, a dynamic measure of pancreatic beta cell function that has been adjusted for the influence of prevailing insulin sensitivity level, in the high GLP-1 prediabetes group treated with linagliptin. A decrease in TyG index in addition to the increase in insulinogenic and oral disposition indices following DPP-IV inhibition in high GLP-1 of the prediabetes subjects represents an improvement in their insulin sensitivity and beta cell function respectively. Preclinical data has demonstrated DPP-IV inhibitors inhibit apoptosis and promote beta cell proliferation thereby increasing beta cell mass (40). The present study highlighted the beneficial effect of DPP-IV inhibitors in improving beta cell function in prediabetes, thus potentially halting the progression to T2D.

In general, linagliptin therapy in both prediabetes and T2D subjects were well tolerated. There was no incidence of hypoglycemia reported. There were also no significant changes in the laboratory parameters at the end of the study. Overall, this is consistent with the safety and tolerability profiles reported (3, 12, 41).

There are several strengths in our study. Notably this is the first study that looked into the endogenous GLP-1 levels and its effects on the subsequent DPP-IV inhibition among prediabetes and T2D populations. In addition, the various clinical and laboratory parameters between the high GLP-1 and low GLP-1 groups in both population at baseline were indistinguishable. Furthermore, the numbers and distribution of pre-existing OGLDs in the T2D population were comparable and they were on a stable dose for at least 3 months. Specifically, our study recruited prediabetes cohort which provides new insights into the role of endogenous GLP-1 levels on glucose metabolism, insulin sensitivity and beta cell function in the presence of DPP-IV inhibition. We also measured total GLP-1 and not the active, intact GLP-1 (7-36 amide) levels. Measurement of intact GLP-1 is compromised by its concentration below the detection limit of the assay due to its extremely rapid degradation by DPP-IV enzyme (1). Therefore, total GLP-1 (comprising both intact GLP-1 and its primary metabolite, GLP-1 9-36 amide) acts as a better indicator of the overall secretory response.

Notwithstanding the strength of the study, the main limitation includes firstly the T2D population recruited were not all newly diagnosed. Only 8 out of 22 T2D subjects were drug naïve. The remaining T2D subjects were on either one, two or three OGLDs. In this respect metformin has been shown to increase endogenous GLP-1 levels. However, this effect is minimised as there are comparable numbers of subjects on metformin in either the high or low GLP-1 groups. Furthermore, they were on a stable dose of the drugs for more than 3 months duration. Secondly, the use of OGTT may not allow us to extend our findings to studies using meal challenge tests.

The clinical relevance of the study is primarily to show that diabetic patients responded to DPPIV-inhibitors differently with regards to its clinical effectiveness. Our data proved that having higher endogenous GLP-1 levels resulted in a better glycaemic efficacy with DPP-IV inhibitors compared to those with lower endogenous GLP-1 levels.

The future perspectives would include different doses of DPPIV-inhibitors would need to be administered for different levels of endogenous GLP-1. This might also lead to alteration in the doses of other concomitant anti-diabetic agents prescribed. Future studies that would be able to identify any clinical or biochemical parameters that could determine an individual’s endogenous GLP-1 concentrations without having to measure the actual GLP-1 levels would be of significant practical relevance. It is also interesting to see whether prediabetes with low GLP-1 levels would need a more intensive diabetes prevention programme than prediabetes with higher GLP-1 levels.

In conclusion, treatment with DPP-IV inhibitor resulted in a greater reduction in HbA1c in subjects with high GLP-1 compared to low GLP-1 levels. This improved efficacy is seen in both the prediabetes and T2D populations. Essentially endogenous GLP-1 levels play an important role in determining DPP-IV inhibitor efficacy irrespective of the abnormal glucose tolerance states.

## Data availability statement

The raw data supporting the conclusions of this article will be made available by the authors, without undue reservation.

## Ethics statement

The studies involving human participants were reviewed and approved by Research Ethics Committee of The National University of Malaysia and the Medical Research & Ethics Committee, Ministry of Health Malaysia. The patients/participants provided their written informed consent to participate in this study.

## Author contributions

NK and NS conceived the idea and conceptualized the study. SC collected and analyzed the data. SC drafted the manuscript. NK, SR, KN and NS reviewed the manuscript. All authors contributed to the article and approved the submitted version.

## Funding

This study was supported by a grant from The Universiti Kebangsaan Malaysia Medical Centre (GUP-2017-066) and a grant from the Malaysian Endocrine & Metabolic Society (L12-MEMS6). The study funders were not involved in the design of the study; the collection, analysis, and interpretation of data; writing the report; and did not impose any restrictions regarding the publication of the report.

## Acknowledgments

We would like to thank all volunteers for their participation in this study and thank laboratory staff members from Chemical Pathology Unit of Hospital Sultan Ismail Johor Bahru and Endocrine Unit of Universiti Kebangsaan Malaysia Medical Centre (UKMMC) for their cooperation and valuable assistance.

## Conflict of interest

The authors declare that the research was conducted in the absence of any commercial or financial relationships that could be construed as a potential conflict of interest.

## Publisher’s note

All claims expressed in this article are solely those of the authors and do not necessarily represent those of their affiliated organizations, or those of the publisher, the editors and the reviewers. Any product that may be evaluated in this article, or claim that may be made by its manufacturer, is not guaranteed or endorsed by the publisher.

## Abbreviations

DPP-IV, Dipeptidyl peptidase-IV; FPG, Fasting plasma glucose; GLP-1, Glucagon-like peptide-1; IS, Insulin sensitivity; LS-mean, Least-squares mean; OGLDs, oral glucose lowering drugs; TyG, Triglyceride-glucose.

## References

[1] Holst JJ. From the incretin concept and the discovery of glp-1 to today's diabetes therapy. Front Endocrinol (2019) 10:260. doi: 10.3389/fendo.2019.00260 PMC6497767 31080438

[2] Ling J Cheng P Ge L Zhang D-H Shi A-C Tian J-H. The efficacy and safety of dipeptidyl peptidase-4 inhibitors for type 2 diabetes: A Bayesian network meta-analysis of 58 randomized controlled trials. Acta Diabetol (2019) 56(3):249–72. doi: 10.1007/s00592-018-1222-z 30242726

[3] Singh-Franco D McLaughlin-Middlekauff J Elrod S Harrington C. The effect of linagliptin on glycaemic control and tolerability in patients with type 2 diabetes mellitus: A systematic review and meta-analysis. Diabetes Obes Metab (2012) 14(8):694–708. doi: 10.1111/j.1463-1326.2012.01586.x 22340363

[4] Guardado-Mendoza R Salazar-Lopez SS Alvarez-Canales M Farfan-Vazquez D Martinez-Lopez YE Jimenez-Ceja LM. The combination of linagliptin, metformin and lifestyle modification to prevent type 2 diabetes (Prellim). A Randomized Clin Trial. Metab Clin Exp (2020) 104:154054. doi: 10.1016/j.metabol.2019.154054 31887309

[5] Alvarez-Canales MFD Salazar-Lopez SS Farfan-Vazquez D Martinez-Lopez YE Gonzalez-Mena JN Jimenez-Ceja LM. Effect of linagliptin on glucose metabolism and pancreatic beta cell function in patients with persistent prediabetes after metformin and lifestyle. Sci Rep (2021) 11(1):8750. doi: 10.1038/s41598-021-88108-8 33888772 PMC8062549

[6] Yabe D Seino Y Fukushima M Seino S. β cell dysfunction versus insulin resistance in the pathogenesis of type 2 diabetes in East asians. Curr Diabetes Rep (2015) 15(6):36–44. doi: 10.1007/s11892-015-0602-9 PMC4420838 25944304

[7] Kim Y Hahn S Oh T Kwak S Park K Cho Y. Differences in the glucose-lowering efficacy of dipeptidyl peptidase-4 inhibitors between asians and non-asians: A systematic review and meta-analysis. Diabetologia (2013) 56(4):696–708. doi: 10.1007/s00125-012-2827-3 23344728

[8] Park H Park C Kim Y Rascati KL. Efficacy and safety of dipeptidyl peptidase-4 inhibitors in type 2 diabetes: Meta-analysis. Ann Pharmacother (2012) 46(11):1453–69. doi: 10.1345/aph.1R041 23136353

[9] Gan S Dawed AY Donnelly LA Nair AT Palmer CN Mohan V. Efficacy of modern diabetes treatments dpp-4i, sglt-2i, and glp-1ra in white and Asian patients with diabetes: A systematic review and meta-analysis of randomized controlled trials. Diabetes Care (2020) 43(8):1948–57. doi: 10.2337/dc19-2419 PMC7372059 33534728

[10] Ito Y Ambe K Kobayashi M Tohkin M. Ethnic difference in the pharmacodynamics-efficacy relationship of dipeptidyl peptidase-4 inhibitors between Japanese and non-Japanese patients: A systematic review. Clin Pharmacol Ther (2017) 102(4):701–8. doi: 10.1002/cpt.692 28378919

[11] Owens DR Swallow R Dugi KA Woerle HJ. Efficacy and safety of linagliptin in persons with type 2 diabetes inadequately controlled by a combination of metformin and sulphonylurea: A 24-week randomized study. Diabetes Med (2011) 28(11):1352–61. doi: 10.1111/j.1464-5491.2011.03387.x 21781152

[12] Del Prato S Barnett AH Huisman H Neubacher D Woerle H-J Dugi KA. Effect of linagliptin monotherapy on glycaemic control and markers of β-cell function in patients with inadequately controlled type 2 diabetes: A randomized controlled trial. Diabetes Obes Metab (2011) 13(3):258–67. doi: 10.1111/j.1463-1326.2010.01350.x 21205122

[13] Taskinen M-R Rosenstock J Tamminen I Kubiak R Patel S Dugi KA. Safety and efficacy of linagliptin as add-on therapy to metformin in patients with type 2 diabetes: A randomized, double-blind, placebo-controlled study. Diabetes Obes Metab (2011) 13(1):65–74. doi: 10.1111/j.1463-1326.2010.01326.x 21114605

[14] Toft-Nielsen M-B Damholt MB Madsbad S Hilsted LM Hughes TE Michelsen BK. Determinants of the impaired secretion of glucagon-like peptide-1 in type 2 diabetic patients. J Clin Endocrinol Metab (2001) 86(8):3717–23. doi: 10.1210/jcem.86.8.7750 11502801

[15] Han SJ Kim HJ Choi S-E Kang Y Lee KW Kim DJ. Incretin secretion and serum dpp-iv activity in Korean patients with type 2 diabetes. Diabetes Res Clin Pract (2010) 89(3):e49–52. doi: 10.1016/j.diabres.2010.06.004 20621378

[16] Lin YH Huang H. Predictors of the efficacy of dipeptidyl peptidase-4 inhibitors in Taiwanese patients with type 2 diabetes mellitus. Diabetes Metab Syndr Obes (2019) 12:2725–33. doi: 10.2147/DMSO.S220180 PMC6935284 31920352

[17] Monami M Cremasco F Lamanna C Marchionni N Mannucci E. Predictors of response to dipeptidyl peptidase-4 inhibitors: Evidence from randomized clinical trials. Diabetes Metab Res Rev (2011) 27(4):362–72. doi: 10.1002/dmrr.1184 21309062

[18] Yagi S Aihara K-I Akaike M Fukuda D Salim HM Ishida M. Predictive factors for efficacy of dipeptidyl peptidase-4 inhibitors in patients with type 2 diabetes mellitus. Diabetes Metab J (2015) 39(4):342–7. doi: 10.4093/dmj.2015.39.4.342 PMC4543199 26301197

[19] Vardarli I Arndt E Deacon CF Holst JJ Nauck MA. Effects of sitagliptin and metformin treatment on incretin hormone and insulin secretory responses to oral and “Isoglycemic” intravenous glucose. Diabetes (2014) 63(2):663–74. doi: 10.2337/db13-0805 24186866

[20] Balas B Baig MR Watson C Dunning BE Ligueros-Saylan M Wang Y. The dipeptidyl peptidase iv inhibitor vildagliptin suppresses endogenous glucose production and enhances islet function after single-dose administration in type 2 diabetic patients. J Clin Endocrinol Metab (2007) 92(4):1249–55. 10.1210/jc.2006-1882 17244786

[21] American Diabetes Association Classification and diagnosis of diabetes: Standards of medical care in diabetes–2020. Diabetes Care (2019) 43(Supplement_1):S14–31. doi: 10.2337/dc20-S002

[22] Friedewald WT Levy RI Fredrickson DS. Estimation of the concentration of low-density lipoprotein cholesterol in plasma, without use of the preparative ultracentrifuge. Clin Chem (1972) 18(6):499–502. 4337382

[23] American Diabetes Association. Postprandial blood glucose. Diabetes Care (2001) 24(4):775–8. doi: 10.2337/diacare.24.4.775 11315848

[24] Matthews DR Hosker JP Rudenski AS Naylor BA Treacher DF Turner RC. Homeostasis model assessment: Insulin resistance and beta-cell function from fasting plasma glucose and insulin concentrations in man. Diabetologia (1985) 28(7):412–9. 10.1007/BF00280883 3899825

[25] Toro-Huamanchumo CJ Urrunaga-Pastor D Guarnizo-Poma M Lazaro-Alcantara H Paico-Palacios S Pantoja-Torres B. Triglycerides and glucose index as an insulin resistance marker in a sample of healthy adults. Diabetes Metab Syndr (2019) 13(1):272–7. doi: 10.1016/j.dsx.2018.09.010 30641711

[26] Yokoyama H Emoto M Fujiwara S Motoyama K Morioka T Komatsu M. Quantitative insulin sensitivity check index and the reciprocal index of homeostasis model assessment are useful indexes of insulin resistance in type 2 diabetic patients with wide range of fasting plasma glucose. J Clin Endocrinol Metab (2004) 89(3):1481–4. doi: 10.1210/jc.2003-031374 15001651

[27] Minh HV Tien HA Sinh CT Thang DC Chen CH Tay JC. Assessment of preferred methods to measure insulin resistance in Asian patients with hypertension. J Clin Hypertens (Greenwich) (2021) 23(3):529–37. doi: 10.1111/jch.14155 PMC8029536 33415834

[28] Tura A Kautzky-Willer A Pacini G. Insulinogenic indices from insulin and c-peptide: Comparison of beta-cell function from ogtt and ivgtt. Diabetes Res Clin Pract (2006) 72(3):298–301. doi: 10.1016/j.diabres.2005.10.005 16325298

[29] Retnakaran R Qi Y Goran MI Hamilton JK. Evaluation of proposed oral disposition index measures in relation to the actual disposition index. Diabetes Med (2009) 26(12):1198–203. doi: 10.1111/j.1464-5491.2009.02841.x 20002470

[30] Chong SC Sukor N Robert SA Ng KF Kamaruddin NA. Fasting and Stimulated Glucagon-Like Peptide-1 Exhibit a Compensatory Adaptive Response in Diabetes and Pre-Diabetes States: A Multi-Ethnic Comparative Study. Frontiers in Endocrinology (2022) 13:961432. doi: 10.3389/fendo.2022.961432 36157456 PMC9501699

[31] Holst JJ Deacon CF. Glucagon-like peptide-1 mediates the therapeutic actions of dpp-iv inhibitors. Diabetologia (2005) 48(4):612–5. doi: 10.1007/s00125-005-1705-7 15759106

[32] Nauck MA Meier JJ. The incretin effect in healthy individuals and those with type 2 diabetes: Physiology, pathophysiology, and response to therapeutic interventions. Lancet Diabetes Endocrinol (2016) 4(6):525–36. doi: 10.1016/S2213-8587(15)00482-9 26876794

[33] Deacon CF Wamberg S Bie P Hughes TE Holst JJ. Preservation of active incretin hormones by inhibition of dipeptidyl peptidase iv suppresses meal-induced incretin secretion in dogs. J Endocrinol (2002) 172(2):355–62. doi: 10.1677/joe.0.1720355 11834453

[34] Calanna S Christensen M Holst JJ Laferrère B Gluud LL Vilsbøll T. Secretion of glucagon-like peptide-1 in patients with type 2 diabetes mellitus: Systematic review and meta-analyses of clinical studies. Diabetologia (2013) 56(5):965–72. doi: 10.1007/s00125-013-2841-0 PMC3687347 23377698

[35] Singh AK. Incretin response in Asian type 2 diabetes: Are indians different? Indian J Endocrinol Metab (2015) 19(1):30–8. doi: 10.4103/2230-8210.146861 PMC4287776 25593823

[36] Cho YM. Incretin physiology and pathophysiology from an Asian perspective. J Diabetes Investig (2015) 6(5):495–507. doi: 10.1111/jdi.12305 PMC4578486 26417406

[37] Daisuke Yabe HK Iwasaki M Seino Y. Why are incretin-based therapies more efficient in East asians? perspectives from the pathophysiology of type 2 diabetes and East Asian dietary habits. Eur Med J (2015) 3(1):57–65.

[38] Son DH Lee HS Lee YJ Lee JH Han JH. Comparison of triglyceride-glucose index and homa-ir for predicting prevalence and incidence of metabolic syndrome. Nutr Metab Carbiovasc Dis (2022) 32(3):596–604. doi: 10.1016/j.numecd.2021.11.017 35090800

[39] Park HM Lee HS Lee Y-J Lee J-H. The triglyceride–glucose index is a more powerful surrogate marker for predicting the prevalence and incidence of type 2 diabetes mellitus than the homeostatic model assessment of insulin resistance. Diabetes Res Clin Pract (2021) 180:109042. doi: 10.1016/j.diabres.2021.109042 34506839

[40] van Genugten RE van Raalte DH Diamant M. Dipeptidyl peptidase-4 inhibitors and preservation of pancreatic islet-cell function: A critical appraisal of the evidence. Diabetes Obes Metab (2012) 14(2):101–11. doi: 10.1111/j.1463-1326.2011.01473.x 21752172

[41] Ning G Bandgar T Hehnke U Lee J Chan JCN. Efficacy and safety of linagliptin in 2681 Asian patients stratified by age, obesity, and renal function: A pooled analysis of randomized clinical trials. Adv Ther (2017) 34(9):2150–62. doi: 10.1007/s12325-017-0595-7 PMC5599450 28819835

